# 2805. Extended-Infusion versus Intermittent-Infusion Piperacillin/tazobactam for High Minimum Inhibitory Concentration *Pseudomonas aeruginosa* Infections

**DOI:** 10.1093/ofid/ofad500.2416

**Published:** 2023-11-27

**Authors:** Carlee R Shifko, Dustin R Carr, Thomas L Walsh, Matthew A Moffa, Nathan R Shively, Derek N Bremmer

**Affiliations:** Allegheny General Hospital, Pittsburgh, Pennsylvania; Allegheny General Hospital, Pittsburgh, Pennsylvania; Allegheny Health Network, Pittsburgh, Pennsylvania; Allegheny Health Network, Pittsburgh, Pennsylvania; Allegheny Health Network, Pittsburgh, Pennsylvania; Allegheny General Hospital, Pittsburgh, Pennsylvania

## Abstract

**Background:**

Extended-infusion (EI) piperacillin/tazobactam, compared to intermittent-infusion (II) piperacillin/tazobactam, has been associated with improved clinical outcomes for both critically ill and febrile neutropenic patients. However, there is a paucity of data on whether these clinical benefits apply to broader populations. Infections involving high minimum inhibitory concentration (MIC) *Pseudomonas aeruginosa* represent an additional group where optimization of pharmacokinetic parameters may impact treatment success.

**Methods:**

Medical records of adults who received II or EI piperacillin/tazobactam for pneumonia and/or bacteremia caused by *Pseudomonas aeruginosa* with MIC 8 or 16 mcg/mL were reviewed. The primary outcome was 30-day all-cause mortality. Hospital and/or intensive care unit (ICU) length of stay (LOS) and recurrence of infection were also recorded. Additionally, an escalation of care composite was assessed by need for new ICU transfer, new vasopressor start, new mechanical ventilation, or rescue antibiotic therapy change. Finally, a safety outcome of acute kidney injury (AKI) was recorded.

**Results:**

A total of 69 patients were included, 31 in the II group and 38 in the EI group. Bacteremia was detected in 19.4% and 23.7% of the II and EI groups, respectively (p = 0.66). Baseline demographics and characteristics of treatment were similar between groups. The most common dosing regimens were 3.375 g IV every 6 hours over 30 minutes and 3.375 g IV every 8 hours over 4 hours. 30-day mortality was 32.3% in the II group and 39.5% in the EI group (p = 0.54). Similarly, hospital LOS (19 versus 15 days, p = 0.27) and ICU LOS (23 versus 15 days, p = 0.15), as well as recurrence (29.4% versus 23.5%, p = 1) and therapy escalation (38.7% versus 47.4%, p = 0.47), were not significantly different between groups. 43.5% of patients in the II group and 40.6% of patients in the EI group developed AKI (p = 0.83).

**Conclusion:**

The present study found no significant difference in outcomes between piperacillin/tazobactam infusion strategies. However, the small sample size and complex patient factors may have limited the comparison.

**Disclosures:**

**Dustin R. Carr, PharmD, BCPS, BCIDP**, Merck: Advisor/Consultant **Thomas L. Walsh, MD**, Accelerate Diagnostics: Advisor/Consultant **Derek N. Bremmer, PharmD, BCIDP**, Thermo Fisher Scientific: Advisor/Consultant

